# Fast Beam Training Technique for Millimeter-Wave Cellular Systems with an Intelligent Reflective Surface

**DOI:** 10.3390/s21144936

**Published:** 2021-07-20

**Authors:** Qasim Sultan, Yeong-Jun Kim, Mohammed-Saquib Khan, Yong-Soo Cho

**Affiliations:** 1 School of Electrical and Electronics Engineering, Chung-Ang University, 84 Heukseok-ro, Dongjak-gu, Seoul 06974, Korea; qasimsultan6@gmail.com (Q.S.); snhk02@gmail.com (M.-S.K.); 2 LG Electronics, Seoul 137-893, Korea; yjkim81@gmail.com

**Keywords:** intelligent reflecting surface, beam training signal, uniform rectangular array

## Abstract

The concept of an intelligent reflecting surface (IRS) has recently emerged as a promising solution for improving the coverage and energy/spectral efficiency of future wireless communication systems. However, as the number of reflecting elements in an IRS increase, the beam training protocol in IRS-assisted millimeter-wave (mmWave) cellular systems requires a large beam training time because it needs to find the best beam pairs for the link between the base station (BS) and the IRS, as well as the link between the IRS and the mobile station (MS). In this paper, a fast beam training technique for IRS-assisted mmWave cellular systems with a uniform rectangular array is proposed for detecting the best beam pairs of BS-IRS and IRS-MS links simultaneously. Two different types of beam training signals (BTSs) are proposed to distinguish simultaneously transmitted beams from the BSs in multi-cell multi-beam environments: the Zadoff–Chu sequence based BTS (ZC-BTS) and m-sequence based BTS (m-BTS). The correlation properties of ZC-BTSs and m-BTSs are analyzed in multi-cell multi-beam environments. In addition, the effect of symbol time offset on the ZC-BTS and m-BTS is analyzed. Finally, simulation results reveal that the proposed technique can significantly reduce the beam training time for IRS-assisted mmWave cellular systems.

## 1. Introduction

As 5G cellular systems are deployed on a commercial scale, technologies for next-generation (6G) communications are being explored to achieve faster and more reliable data transmission. Among these technologies, the intelligent reflective surface (IRS) has garnered significant interest because of its ability to improve spectral and energy efficiencies. The IRS is constructed by planar surfaces consisting of a large number of low-cost passive reflecting elements that are adjustable by a smart controller. These elements introduce phase shifts and amplitude variations of the incident signals so that the incident electromagnetic wave can be directed in the desired directions. Because the IRS eliminates the use of active RF elements, it consumes almost no additional power [1,2,3].

Furthermore, millimeter-wave (mmWave) and terahertz (THz) communications are being investigated because they can yield a significant increase in mobile data traffic for an advanced broadband cellular communication. For mmWave/THz communications, highly directional beamforming antennas are required at both the base station (BS) and mobile station (MS) to compensate for the high attenuation in the mmWave/THz frequency band and extend the transmission range. With a small wavelength of an mmWave/THz frequency, antenna arrays can be easily installed at the MS. The IRS is particularly useful for coverage extension in the mmWave/THz communications that are highly vulnerable to blockage. In this case, the IRS can provide an alternative path between the BS and the MS [4,5,6,7].

Narrow beam transmission and reception are effective for improving the link budget in mmWave/THz frequencies. However, the beam training protocol in a cellular system requires a large amount of training time because it needs to find the best beam pair in the BS-MS link to gain maximum beamforming efficiency. The overhead for beam training in an IRS-assisted mmWave/THz cellular system increases further because the best beam pairs in the BS-IRS-MS link should be found. The overhead increases exponentially when sharp pencil beams generated by a massive number of reflecting elements are used in the IRS. The beam training technique developed for the BS-MS link cannot be directly applied to the BS-IRS link for the best beam pair between the BS and IRS, and the IRS-MS link for the best beam pair between the IRS and MS. Normally, the best beam pair between the BS and the IRS cannot be obtained at the IRS because the IRS simply reflects the received signal. Full RF chains and baseband processing units are required at the IRS for detection of the best beam pair between the BS and the IRS. In addition, the best beam pair between the IRS and the MS cannot be obtained at the MS without the BS because the IRS consists of passive reflecting elements. Unlike the BS with an active power source [8,9,10,11,12], the IRS cannot transmit training signals. Consequently, it is difficult to find the best beam pair separately for the BS-IRS link and IRS-MS link. In this paper, we propose a fast beam training technique that can simultaneously detect the best beam pairs (BS-IRS-MS) in IRS-assisted cellular systems.

The authors in [13] proposed a fast beamforming algorithm for multi-group multi-cast beamforming design in large-scale wireless systems. The alternating direction method of multipliers (ADMM) and convex-concave procedure (CCP) based low complexity and high performance algorithm is proposed in this paper considering channel state information (CSI) is known at the transmitter. However, since our fast 3D beamforming technique is developed for beam search in the initialization stage, it is assumed that no information on CSI is available at the BS in our paper. In [14], iterative procedure with variable step sizes was proposed with distributed antenna systems for fast beamforming. This paper proposed the creation of virtual subarray using two or more distributed antennas. The virtual array can align transmitted signal phases at the intended user. The major drawback for this technique is about coordination of sources (information sharing, timing synchronization, carrier synchronization) among elements of virtual array. However, this problem does not occur in our case because the beams are transmitted from the same source (3D beamformer). The beams transmitted from multiple ULAs at the BS are all synchronized in time and frequency. The authors in [15] proposed a statistics based fast initial access procedure. This paper also proposed an online implementation method which acquires the MS statistics and adapts the initial access scanning procedure. However, the past user statistics of MS behavior is not exploited in our fast 3D beamforming technique. In our beamforming technique, Tx and Rx beams are determined by calculating the correlation value between the received signal at the MS and reference signal.

In [16], the concept of RF focus (RFocus) including 2D surface with simple elements and a software-based controller was introduced. This surface reflects the signal and properties of surface can be controlled using a software controller. It focuses the RF power in the desired direction improving the link-budget at the receiver. The RFocus surface can be manufactured as an inexpensive thin wallpaper, requiring no wiring. In [17], the authors introduced the concept of massive backscatter communication with programmable meta-surfaces at the transmitter, which modulates the propagation environment of stray ambient waves. The resulting wave control enables focusing and multi-channel schemes, which allowed them to demonstrate unprecedented data rates on the order of hundreds of kbps and communication security—without requiring an active radiofrequency chain, energy, and spectral resources to generate a carrier signal. In [18], an architecture based on time-domain digital-coding meta-surface was proposed for new wireless communication systems. By dynamically modulating the local phase of surface reflectivity in the meta-surface-based system, the authors can achieve accurate control of different harmonics in a highly programable and dynamic fashion. Compared to conventional systems, the hardware complexity of the metasurface-based system is greatly simplified without degrading the system performance. In [19], the concept of intelligent wall was proposed as an autonomous part of a smart indoor environment for cognitive wireless networks. Artificial neural networks were employed in the cognitive engine to make each node capable of learning from past experiences. The smart environment can react to the immediate demands of an indoor wireless system, control radio coverage, and, consequently, influence overall system performance. In [20], the authors proposed programmable coding meta-surfaces to directly transmit “0”s and “1”s into space, eliminating the requirement for analog-digital convertor and a series of active and passive microwave devices. The authors built the prototype to validate the new architecture experimentally, which may find promising applications where information security is highly demanded. In [21], it was shown that optimal channel diversity can be achieved by physically shaping the propagation medium with a reconfigurable meta-surface placed inside a random environment. Enhanced wireless image transmission was demonstrated in an office room by reducing channel cross-talk and obtaining orthogonality of wireless channels with a reconfigurable meta-surface.

Various beamforming optimization techniques have also been proposed for IRS-assisted communication systems [22,23,24,25,26,27,28,29]. Recently, a fast beam training technique for IRS-assisted multi-user communications was proposed that could significantly reduce the beam training time for the IRS-MS link using multi-beam transmission and multi-beam codebook design [30]. The optimal IRS beam directions were detected with a high probability, without compromising the IRS passive performance for data transmission. Although the technique is practical and effective in IRS-assisted communication systems, it can be applied to single cell environments. In multi-cell environments, beam training signals (BTSs) are transmitted simultaneously from neighboring cells to the MSs in their cells during the initialization period. Therefore, BTSs transmitted from neighboring cells act as interference, particularly for MSs located at the cell boundary. If the MS cannot identify the source BS of the BTS from the received signal, it cannot find the best beam direction for the serving BS with the IRS. In [30], the beam training technique was developed for the IRS-MS link, assuming that the BS-IRS link (beam pair) had already been established.

In this paper, a fast beam training technique is proposed to simultaneously detect the best beam pairs (BS-IRS-MS) in mmWave cellular systems with an IRS. In the proposed technique, it is assumed that mmWave beamforming is performed at the BS, IRS, and MS, all using a uniform rectangular array (URA). The URA is considered to be a set of uniform linear arrays (ULAs). The URA at the BS is divided into ULAs that transmit beams in different directions simultaneously. The URA at the IRS is also divided into ULAs, which reflect the incoming signals in different directions. Because multiple beams are received in multi-cell environments, the MS must be capable of identifying its cell ID (CID) and beam ID (BID) from the received signals. In this paper, two BTSs are proposed to facilitate joint detection of the CID and BID at the MS: Zadoff–Chu sequence based BTS (ZC-BTS) and m-sequence based BTS (m-BTS) [31]. The properties of the ZC-BTS and m-BTS are analyzed in a multi-cell environment, where time delays exist among the signals received from neighboring BSs. Simulation results reveal that the proposed BTSs can be used to find the best beam pairs (BS-IRS-MS) in mmWave cellular systems with a significant reduction in beam training time.

The remainder of this paper is organized as follows: Section 2 describes the proposed beam training technique for IRS-assisted cellular systems. In Section 3, two different types of BTSs are discussed and their correlation properties are analyzed in a multi-cell multi-beam environment. In Section 4, the performance of the proposed technique is evaluated using a simple IRS-assisted cellular system model. Conclusions are drawn in Section 5.

## 2. Proposed Beam Training Technique for IRS-Assisted Cellular Systems

The beam training is performed in all possible directions during the initialization period for mmWave cellular systems because a BS has no information about the position of an MS. The beam training time increases proportionally with the number of beams in mmWave cellular systems when exhaustive search is used [32,33]. The beam training time increases further for IRS-assisted cellular systems because the best beam pairs should be found not only for the IRS-MS link but also for the BS-IRS link. Because the IRS consists of passive reflecting elements, the best beam pairs in the BS-IRS-MS link should be found through the signals transmitted from the active power source in the BS. The beam training time in IRS-assisted cellular systems increases proportionally with the product of the number of transmitted beams at the BS, number of reflecting beams at the IRS, and number of received beams at the MS when an exhaustive search is used. This long processing time will create significant overhead for IRS-assisted cellular systems. In this section, we describe a beam training technique that can simultaneously detect the best beam pairs (BS-IRS-MS) in an IRS-assisted cellular system with URAs.

**Figure 1:**
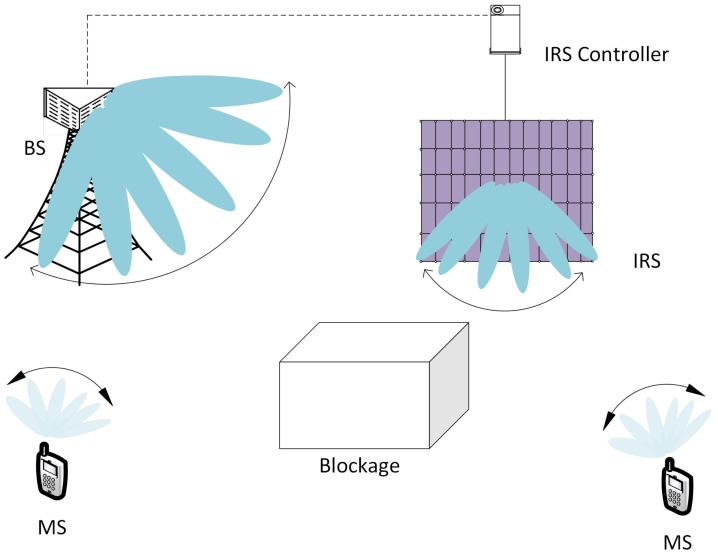
Beam training for an IRS-assisted cellular system.

**Figure 2:**
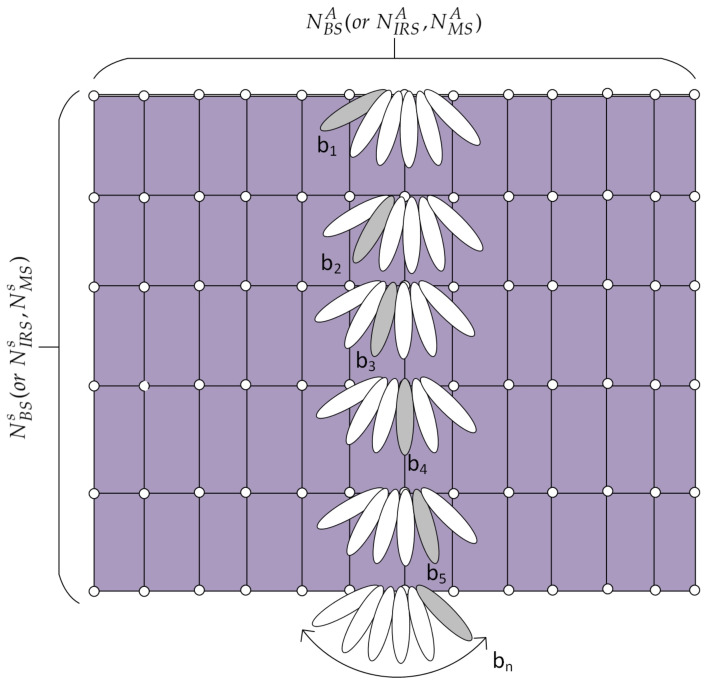
Uniform rectangular arrays at the BS, IRS, and MS.

Figure 1 presents a conceptual schematic diagram for beam training in IRS-assisted cellular systems. Some users are served by the BS directly. However, the line-of-sight (LOS) link between the BS and MS can be blocked owing to some obstacles. In this case, an IRS can provide an alternative path between the BS and MS and keep the MS connected to the network. In this paper, we focus on a beam training technique for the BS-IRS-MS link because beam training techniques for the BS-MS link are available. In the proposed technique, the URA at the BS is divided into $N_{BS}^{s}$ subarrays (ULAs), with each subarray consisting of $N_{BS}^{A}$ antenna elements. As shown in Figure 2, beams are transmitted from $N_{BS}^{s}$ subarrays simultaneously. Because the source of the signal should be identified in a multi-cell environment with multiple beams, the CID and BID are incorporated in the design of the BTS. Here, $\theta_{BS}^{b}$ denotes the direction (angle) of the beam with BID *b*. The total number of beam directions at the BS is given by $N_{BS}^{b}$. If the number of beams at the BS is greater than the number of subarrays $N_{BS}^{b}>N_{BS}^{s}$, this transmission process is repeated *u* times, i.e., $N_{BS}^{b}=u\times N_{BS}^{s}$. Here, *u* is an integer. The design technique for the BTS is described in Section 3.

The IRS is a passive device that reflects the signal received from the BS. The beam direction of the reflected signal can be adjusted by changing the phase shifters in a passive manner. An IRS controller is used to control the beam direction and exchange information with the BS via a separate reliable link. As shown in Figure 2, the URA at the IRS is divided into $N_{IRS}^{s}$ horizontal subarrays, with each subarray consisting of $N_{IRS}^{A}$ reflecting elements, similar to the URA at the BS. Each subarray at the IRS reflects the signals (BTSs) received from the BS in different beam directions $\theta_{IRS}^{b}$. The reflecting beam direction at the IRS changes sequentially over different symbols. Thus, the BID of the IRS is given by the symbol index, unlike the BID of the BS. The total number of reflecting beam directions is given by $N_{IRS}^{b}$. If $N_{IRS}^{b}>N_{IRS}^{s}$; this process is repeated *v* times, i.e., $N_{IRS}^{b}=v\times N_{IRS}^{s}$. Here, *v* is an integer. If the number of subarrays is equal to the number of beams, only one round of transmission is required.

The MS receives the signals reflected from the IRS and performs correlation with the reference BTSs to find the best beam pairs (BS-IRS-MS) as well as the CID. As shown in Figure 2, the URA at the MS is also divided into $N_{MS}^{s}$ subarrays, with each subarray consisting of $N_{MS}^{A}$ elements. Each subarray receives signals in different beam directions i.e., $\theta_{MS}^{b}$. Here, the total number of received beam directions is given by $N_{MS}^{b}$. If $N_{MS}^{s}$ is smaller than $N_{MS}^{b}$, the beam search operation at the MS is repeated *w* times, i.e., $N_{MS}^{b}=w\times N_{MS}^{s}$. If the number of subarrays at the MS is identical to the number of beams at the MS, only one cycle is required. Thus, the total number of beam scans required for the BS-IRS-MS link in the proposed technique is given by $u\times \left(v+N_{IRS}^{b}\right)\times w$. In the proposed technique, the best BID and CID of the BS are determined at the MS by correlating the received signal with reference BTSs because the BTSs are transmitted simultaneously from the BS. The best beam pair for the IRS and MS is determined at the MS by comparing the received signal power over time (symbol) and antenna subarrays, respectively. Here, it is assumed that the distance between the BS and IRS and the distance between the IRS and MS are considerably larger than the spacing between antenna elements in the URA (BS, IRS, and MS). In this case, the subarrays transmitting/receiving beams in different directions can be assumed to be located at the same position (height). The procedure of the proposed beam training technique for the BS-IRS-MS link can be summarized as follows:

First, the BS transmits multiple beams simultaneously in all possible directions using a set of ULAs. The BTS transmitted from each beam contains CID and BID so that they can be detected at the MS in a multi-cell multi-beam environment. Second, the IRS reflects the signal received from the BS in different beam directions. The reflecting beam direction at the IRS changes sequentially over time. Third, the MS receives the signals reflected from the IRS using a set of ULAs. The ULAs receive signals simultaneously from different beam directions. The BID and CID of the BS are determined by correlating the received signal with the reference BTSs, whereas the BIDs of the IRS and MS are determined by comparing the received signal power over time.

## 3. Proposed BTSs for IRS-Assisted Cellular Systems

When the proposed technique is used for beam training in an IRS-assisted cellular system, the received signal at the MS in the BS-IRS-MS link can be expressed as follows:

(1) $$\begin{array}{c} \begin{array}{c} y\left(n\right)=y^{\bar{c},\bar{b}}\left(n\right)+y^{\bar{c},b}\left(n\right)+y^{c,b}\left(n\right)+\eta \left(n\right) \end{array} \end{array}$$

where

$$\begin{array}{c} \begin{array}{c} y^{\bar{c},\bar{b}}\left(n\right)=\gamma_{BS}^{\bar{c},\bar{b}}\gamma_{IRS}^{tx}\gamma_{MS}^{rx}h_{BS-MS}^{\bar{c},\bar{b}}q^{\bar{c},\bar{b}}\left(n-\Delta_{}^{\bar{c},\bar{b}}\right)\\ y^{\bar{c},b}\left(n\right)=\sum_{b=0}^{N_{BS}^{b}-1}\gamma_{BS}^{\bar{c},b}\gamma_{IRS}^{tx}\gamma_{MS}^{rx}h_{BS-MS}^{\bar{c},b}\left(n\right)\times q^{\bar{c},b}\left(n-\Delta_{}^{\bar{c},b}\right)\\ y^{c,b}\left(n\right)=\sum_{c=0}^{N_{c}-1}\sum_{b=0}^{N_{BS}^{b}-1}\gamma_{BS}^{c,b}\gamma_{IRS}^{tx}\gamma_{MS}^{rx}h_{BS-IRS}^{c,b}\left(n\right)\times q^{c,b}\left(n-\Delta_{}^{c,b}\right) \end{array} \end{array}$$

where $y^{\bar{c},\bar{b}}$ represents the signal received from the serving BS with the best beam. Here, *c* denotes the CID, ranging from zero to $N_{c}-1$. $N_{c}$ is the number of neighboring cells. $\bar{c}$ and $\bar{b}$ denote the reference CID and reference BID, respectively. In addition, $y^{\bar{c},b}$ is the interference signal received from the serving BS with different BIDs. $y^{c,b}$ represents the interference signal received from the neighboring BS with BID *b*. Finally, η represents the noise term. Here, $\gamma_{BS}^{c,b},\gamma_{MS}^{rx},\gamma_{IRS}^{tx},h_{BS-MS}^{c,b},$ and $q^{c,b}$ are the transmit beamforming gain of the *b*th beam of the *c*th BS, receive beamforming gain of the MS, beamforming gain of the IRS, effective channel in the BS-IRS-MS link, and BTS transmitted from the BS, respectively. The channel is assumed to be time-invariant and frequency-flat. In addition, $\Delta_{}^{c,b}$ denotes the propagation delay between the BS with (c,b) and MS in the BS-IRS-MS link. As the distance between the BS and IRS and the distance between the IRS and MS are considerably larger than the antenna spacing, $\Delta_{}^{c,b}$ can be simply expressed as $\Delta_{}^{c}$. Note that the BIDs for IRS and MS are not considered in (1) because they can be easily determined by finding the position of the peak value in the received signal power at the MS over time and antenna subarrays, respectively.

In the proposed technique, multiple BTSs are simultaneously transmitted from the URA of the BS to reduce the beam training time in the BS-IRS-MS link. The CID and BID are incorporated in the design of the BTS so that they can be detected at the MS in a multi-cell multi-beam environment. The BTSs that are not matched with the reference CID and the reference BID will act as an interference. To facilitate joint detection of the CID and BID with minimum inter-beam and inter-cell interferences, two BTSs are proposed for IRS-assisted cellular systems.

### 3.1. Zadoff–Chu Sequence Based BTS (ZC-BTS)

Owing to a good correlation property and low peak-to-average power (PAPR), the ZC sequence has been widely used to design synchronization signals in various applications, including the primary synchronization signal (PSS) in LTE and random access in 5G-NR-based cellular systems [34,35]. In the proposed BTS, beams are transmitted simultaneously from the BS subarrays to find the best beam pairs in the BS-IRS-MS link. The CID and BID are mapped to a ZC sequence of a prime length so that they can be detected in a multi-cell multi-beam environment as follows:

(2) $$\begin{array}{c} q_{zc}^{c,b}\left(n\right)=e^{\frac{j\pi r^{c}\left(n+zb\right)\left(n+zb+1\right)}{L}} \end{array}$$

where $1<r^{c}<L$ and $\gcd\left(r,L\right)=1$. Here, $q_{zc}^{c,b}$ denotes the ZC-BTS with CID *c* and BID *b*. “gcd” stands for the greatest common divisor. In addition, $r^{c}$, *z*, and *L* denote the root index of the ZC sequence carrying CID *c*, cyclic shift spacing of the ZC sequence, and sequence length, respectively. The ZC sequence has the property of zero autocorrelation and a constant envelope. The ZC sequences with different root indices have a cross-correlation of $1/\sqrt{L}$. The maximum number of CIDs for the ZC-BTS is given by L−1. Because multiple ZC-BTSs are simultaneously transmitted from the subarrays of the BS in the proposed technique, a correlation operation is performed at the MS to find the best CID and BID of the BS in the BS-IRS-MS link. The correlation between the received signal and reference ZC-BTS is given by:

(3) $$\begin{array}{c} C_{ZC-BTS}^{c,b}\left(m\right)=\sum_{n=0}^{L-1}y\left(n\right){\left(q_{zc,ref}^{\bar{c},\bar{b}}\left(n-m\right)\right)}^{*} \end{array}$$

(4) $$C_{ZC-BTS}^{c,b}=\Sigma_{ZC-BTS}+Y_{ZC-BTS}+\Lambda_{ZC-BTS}+\Omega_{ZC-BTS}$$

where

$$\begin{array}{c} \begin{array}{c} \Sigma_{ZC-BTS}\left(m\right)=\sum_{n=0}^{L-1}y^{\bar{c},\bar{b}}\left(n\right){\left(q_{zc,ref}^{\bar{c},\bar{b}}\left(n-m\right)\right)}^{*}\\ Y_{ZC-BTS}\left(m\right)=\sum_{n=0}^{L-1}y^{\bar{c},b}\left(n\right){\left(q_{zc,ref}^{\bar{c},\bar{b}}\left(n-m\right)\right)}^{*}\\ \Lambda_{ZC-BTS}\left(m\right)=\sum_{n=0}^{L-1}y^{c,b}\left(n\right){\left(q_{zc,ref}^{\bar{c},\bar{b}}\left(n-m\right)\right)}^{*}\\ \Omega_{ZC-BTS}\left(m\right)=\sum_{n=0}^{L-1}\eta {\left(q_{zc,ref}^{\bar{c},\bar{b}}\left(n-m\right)\right)}^{*} \end{array} \end{array}$$

Here, $q_{zc,ref}^{\bar{c},\bar{b}}$ denotes the reference ZC-BTS. In (4), $\Sigma_{ZC-BTS}$ represents the autocorrelation value when the received signal is matched with the reference ZC-BTS $\left(c=\bar{c},b=\bar{b}\right)$. The terms inside the summation can be expressed as:

(5) $$\begin{array}{c} \begin{array}{c} \sum_{n=0}^{L-1}q_{zc}^{\bar{c},\bar{b}}\left(n-\Delta^{\bar{c}}\right){\left(q_{zc,ref}^{\bar{c},\bar{b}}\left(n-m\right)\right)}^{*}\\ =\sum_{n=0}^{L-1}e^{\frac{j\pi r^{\bar{c}}\left(n-\Delta^{\bar{c}}+z\bar{b}\right)\left(n-\Delta^{\bar{c}}+z\bar{b}+1\right)}{L}}{\left(e^{\frac{j\pi r^{\bar{c}}\left(n-m+z\bar{b}\right)\left(n-m+z\bar{b}+1\right)}{L}}\right)}^{*}\\ =e^{\frac{j\pi r^{\bar{c}}}{L}\left[{\left(\Delta^{\bar{c}}\right)}^{2}-2z\bar{b}\Delta^{\bar{c}}-\Delta^{\bar{c}}-m^{2}+2z\bar{b}m+m\right]}\\ \;\;\;\;\times e^{j\pi \left(2m-2\Delta^{\bar{c}}\right)\left(L-1\right)/L}\cdot \frac{\sin\pi \left(2m-2\Delta^{\bar{c}}\right)}{\sin\left(\pi \left(2m-2\Delta^{\bar{c}}\right)/L\right)}. \end{array} \end{array}$$

The correlation is performed at the MS by shifting time lag of the reference ZC-BTS. It can be observed from (5) that the correlation value is maximum when the symbol timing offset (STO) between propagation delay $\Delta^{\bar{c}}$ and time lag *m* is zero. The correlation decreases with an increase in the STO in the sinc-like pattern. In (4), $Y_{ZC-BTS}$ represents the interference term caused by the correlation when the received signal has the same CID but a different BID $\left(c=\bar{c},b\neq \bar{b}\right)$. The terms inside the summation are given in (6),

(6) $$\begin{array}{c} \begin{array}{c} \sum_{n=0}^{L-1}q_{zc}^{\bar{c},b}\left(n-\Delta^{\bar{c}}\right){\left(q_{zc,ref}^{\bar{c},\bar{b}}\left(n-m\right)\right)}^{*}\\ =\sum_{n=0}^{L-1}e^{\frac{j\pi r^{\bar{c}}\left(n-\Delta^{\bar{c}}+zb\right)\left(n-\Delta^{\bar{c}}+zb+1\right)}{L}}{\left(e^{\frac{j\pi r^{\bar{c}}\left(n-m+z\bar{b}\right)\left(n-m+z\bar{b}+1\right)}{L}}\right)}^{*}\\ =\underset{q_{zc}^{\bar{c},b}\left(0\right)}{\underset{︸}{e^{\frac{j\pi r^{\bar{c}}}{L}\left[zb(zb+1)\right]}}}\;\cdot \;\underset{q_{zc}^{\bar{c},\bar{b}}{\left(0\right)}^{*}}{\underset{︸}{e^{\frac{j\pi r^{\bar{c}}}{L}\left[z\bar{b}\left(z\bar{b}+1\right)\right]}}}\times \\ e^{\frac{j2\pi r^{\bar{c}}}{L}\left[{\left(\Delta^{\bar{c}}\right)}^{2}-2zb\Delta^{\bar{c}}-\Delta^{\bar{c}}-m^{2}+2z\bar{b}m+m\right]}\sum_{n=0}^{L-1}e^{\frac{j2\pi }{L}n\left[r^{\bar{c}}\left(zb-z\bar{b}-\Delta^{\bar{c}}+m\right)\right]}\\ =\left\{\begin{array}{c} q_{zc}^{\bar{c},b}\left(0\right)q_{zc}^{\bar{c},\bar{b}}{\left(0\right)}^{*}\;\cdot \;L\;\;\mathrm{if}\;\xi^{\left(\bar{c},z,b,\bar{b},m,\Delta^{\bar{c}}\right)}=\sigma L\\ 0\;\;\;\;\;\;\;\;\mathrm{otherwise} \end{array}\right. \end{array} \end{array}$$

where

(7) $$\begin{array}{c} \xi^{\left(\bar{c},z,b,\bar{b},m,\Delta^{\bar{c}}\right)}=r^{\bar{c}}\left[zb-z\bar{b}-\Delta^{\bar{c}}+m\right]. \end{array}$$

Here, σ is an arbitrary integer. According to (6), the correlation becomes high when the following condition is satisfied:

(8) $$\begin{array}{c} r^{\bar{c}}\left[zb-z\bar{b}-\Delta^{\bar{c}}+m\right]=\sigma L. \end{array}$$

In this case, the correlation peak occurs at an incorrect position, which causes a detection error. Thus, the cyclic shift spacing (z) must be selected to avoid the following condition:

(9) $$\begin{array}{c} z=\frac{\sigma L+r^{\bar{c}}\left(\Delta^{\bar{c}}-m\right)}{r^{\bar{c}}\left(b-\bar{b}\right)},z>0 \end{array}$$

The maximum value of the cyclic shift spacing can be obtained as follows:

(10) $$\begin{array}{c} z_{\max}=\left\lfloor \frac{L}{N_{BS}^{b}}\right\rfloor \end{array}$$

If the value of the cyclic shift spacing is properly selected to avoid the above ambiguity condition, the correlation value will be zero, resulting in negligible inter-beam interference. However, as the maximum STO value increases, the value of cyclic shift spacing must be increased to avoid ambiguity, resulting in reduction of the available IDs. In (4), $\Lambda_{ZC-BTS}$ represents the interference term caused by cross-correlation when the received signal has a different CID $\left(c\neq \bar{c}\right)$. The correlation value is obtained in (11) using the Gaussian sum property [36].

(11) $$\begin{array}{c} \begin{array}{c} \sum_{n=0}^{L-1}q_{zc}^{c,b}\left(n-\Delta^{c}\right){\left(q_{zc,ref}^{\bar{c},\bar{b}}\left(n-m\right)\right)}^{*}=\sum_{n=0}^{L-1}e^{\frac{j\pi r^{c}\left(n-\Delta^{c}+zb\right)\left(n-\Delta^{c}+zb+1\right)}{L}}{\left(e^{\frac{j\pi r^{\bar{c}}\left(n-m+\bar{z}\bar{b}\right)\left(n-m+\bar{z}\bar{b}+1\right)}{L}}\right)}^{*}\\ =q_{zc}^{c,b}\left(0\right){\left[q_{zc}^{\bar{c},\bar{b}}\left(0\right)\right]}^{*}\;\cdot \;e^{\frac{j\pi }{L}r^{c}\left({\left(\Delta^{c}\right)}^{2}-2zb\Delta^{c}-\Delta^{c}\right)}\;\cdot \;e^{-\frac{j\pi }{L}r^{\bar{c}}\left(m^{2}-2z\bar{b}m-m\right)}e^{\frac{-j\pi \chi }{L}}\;\cdot \;\sqrt{L}\;\cdot \;\Theta \end{array} \end{array}$$

where

$$\begin{array}{c} \begin{array}{c} \chi =\left(r^{c}zb-r^{\bar{c}}\bar{z}\bar{b}-r^{\bar{c}}\Delta^{c}+r^{\bar{c}}m\right)\left(\left(r^{c}zb-r^{\bar{c}}\bar{z}\bar{b}-r^{\bar{c}}\Delta^{c}+r^{\bar{c}}m\right){\left(r^{c}-r^{\bar{c}}\right)}^{-1}+1\right)\\ \Theta =\left\{\begin{array}{c} \kappa \frac{1-j^{N}}{1-j},r^{c}>r^{\bar{c}}\\ \kappa \frac{1+j^{N}}{1+j},r^{c}<r^{\bar{c}} \end{array}\right.\\ \kappa =\langle \frac{\alpha \left|r_{c\bar{c}}\right|}{L}\rangle e^{-j\frac{2\pi b_{c\bar{c}}g\gamma^{2}}{L}}\\ \gcd\left(r^{\left(c\right)},L\right)=1,\gcd\left(r^{\left(\bar{c}\right)},L\right)=1,\gcd\left(r_{c\bar{c}},L\right)=1\\ g=\left(L+1\right)/2,\;\gamma =\left(L-1\right)/2,b_{c\bar{c}}=r^{c}-r^{\bar{c}} \end{array} \end{array}$$

Here, 〈 〉 and $\bar{z}$ denote the Jacobi symbol [36] and cyclic shift spacing of the reference BTS, respectively. According to (11), the cross-correlation is bounded by $\sqrt{L}$. Finally, $\Omega_{ZC-BTS}$ represents the noise term correlated with the reference ZC-BTS.

In (6) and (11), it can be observed that the inter-beam and inter-cell interferences are small when the proposed ZC-BTS is used. The actual interference power received at the MS will be negligible because the beamforming gains in (1) are small when the transmit and receive beams are not aligned. According to (5), the maximum correlation is obtained when the received signal is matched with the reference ZC-BTS. In this case, the beamforming gains in (1) are maximized because the transmitted and received beams are aligned. Thus, the best BID and CID can be found using the ZC-BTS if the cyclic shift spacing is adjusted to avoid the ambiguity condition in (9).

### 3.2. M-Sequence Based BTS (m-BTS)

The m-sequence is widely used for preamble design in cellular systems, such as the PSS and secondary synchronization signal (SSS) of 5G-NR and SSS of LTE, owing to its good autocorrelation properties [35]. However, the m-sequence is not used for applications requiring different sequence generation because of its poor cross-correlation property. The Gold sequence (GS), generated by selecting preferred pairs of m-sequences and their combinations, is often used for applications requiring a large set of sequence generation because of its enhanced cross-correlation property. In this paper, a new signal (m-BTS) based on an m-sequence is proposed for fast beam training in IRS-assisted mmWave cellular systems. The m-BTS is generated as follows:

(12) $$\begin{array}{c} q_{m}^{c,b}\left(n\right)=S\left(n+d_{c}\right){\left(S\left(n+d_{b}\right)\right)}^{*} \end{array}$$

where $S={\left(1/L\right)}^{1/2}\sum_{n=0}^{L-1}s_{n}^{}e^{-j2\pi wn/L},L=2^{n}-1,0\leq d_{c},d_{b}<L,and\;d_{c}\neq d_{b}$.

Here, *S* denotes the DFT of m-sequence *s*. Different values of cyclic shifts $\left(d_{c},d_{b}\right)$ corresponding to CID and BID are used to generate $q_{m}^{c,b}$. $q_{m}^{c,b}$ is obtained by multiplying two sequences $\left(S,S^{*}\right)$, which are the DFTs of the m-sequences with different cyclic shifts. The number of IDs that can be generated by m-BTS is given by $\left(L-1\right)L$. Here, *L* denotes the length of the m-sequence. The correlation between the received signal and the reference m-BTS is given by

(13) $$\begin{array}{c} \begin{array}{c} C_{m-BTS}^{c,b}\left(m\right)=\sum_{n=0}^{L-1}y\left(n\right){\left(q_{m,ref}^{\bar{c},\bar{b}}\left(n-m\right)\right)}^{*} \end{array} \end{array}$$

(14) $$\begin{array}{c} \begin{array}{c} C_{m-BTS}^{c,b}=\Sigma_{m-BTS}+Y_{m-BTS}+\Lambda_{m-BTS}+\Omega_{m-BTS} \end{array} \end{array}$$

where

$$\begin{array}{c} \begin{array}{c} \Sigma_{m-BTS}\left(m\right)=\sum_{n=0}^{L-1}y^{\bar{c},\bar{b}}\left(n\right){\left(q_{m,ref}^{\bar{c},\bar{b}}\left(n-m\right)\right)}^{*}\\ Y_{m-BTS}\left(m\right)=\sum_{n=0}^{L-1}y^{\bar{c},b}\left(n\right){\left(q_{m,ref}^{\bar{c},\bar{b}}\left(n-m\right)\right)}^{*}\\ \Lambda_{m-BTS}\left(m\right)=\sum_{n=0}^{L-1}y^{c,b}\left(n\right){\left(q_{m,ref}^{\bar{c},\bar{b}}\left(n-m\right)\right)}^{*}\\ \Omega_{m-BTS}\left(m\right)=\sum_{n=0}^{L-1}\eta {\left(q_{m,ref}^{\bar{c},\bar{b}}\left(n-m\right)\right)}^{*} \end{array} \end{array}$$

Here, $q_{m,ref}^{\bar{c},\bar{b}}$ denotes the reference m-BTS. In (14), $\Sigma_{m-BTS}$ represents the autocorrelation value when the received signal is matched with the reference m-BTS $\left(c=\bar{c},b=\bar{b}\right)$. In this case, the terms inside the summation can be rewritten as

(15) $$\begin{array}{c} \begin{array}{c} \sum_{n=0}^{L-1}q_{m}^{c,b}\left(n-\Delta^{\bar{c}}\right){\left(q_{m,ref}^{\bar{c},\bar{b}}\left(n-m\right)\right)}^{*}\\ =\frac{1}{L}\sum_{n=0}^{L-1}\left(S\left(n+d_{c}-\Delta^{\bar{c}}\right){\left(S\left(n+d_{b}-\Delta^{\bar{c}}\right)\right)}^{*}\right){\left(S\left(n+d_{\bar{c}}-m\right){\left(S\left(n+d_{\bar{b}}-m\right)\right)}^{*}\right)}^{*}\\ =\frac{1}{L}\sum_{n=0}^{L-1}\left(\sum_{w_{0}=0}^{L-1}\sum_{w_{1}=0}^{L-1}s_{w_{0}}^{}e^{-j2\pi w_{0}\left(n+{d^{\prime }}_{c}\right)/L}s_{w_{1}}^{}e^{j2\pi \left(w_{1}\right)\left(n+{d^{\prime }}_{b}\right)/L}\right)\\ \;\;\;\;\;\;\;\times {\left(\sum_{w_{2}=0}^{L-1}\sum_{w_{3}=0}^{L-1}s_{w_{2}}^{}e^{-j2\pi w_{2}\left(n+{d^{\prime }}_{\bar{c}}\right)/L}s_{w_{3}}^{}e^{j2\pi w_{3}\left(n+{d^{\prime }}_{\bar{b}}\right)/L}\right)}^{*} \end{array} \end{array}$$

where

$$\begin{array}{c} \begin{array}{c} {d^{\prime }}_{c}=d_{c}-\Delta^{\bar{c}}\\ {d^{\prime }}_{b}=d_{b}-\Delta^{\bar{c}}\\ {d^{\prime }}_{\bar{c}}=d_{c}-m\\ {d^{\prime }}_{\bar{b}}=d_{b}-m \end{array} \end{array}$$

If ${\left(n_{1}-n_{0}\right)}_{\%L}=\delta_{0}$ and ${\left(n_{3}-n_{2}\right)}_{\%L}=\delta_{1}$, (15) can be expressed as

(16) $$\begin{array}{c} \begin{array}{c} \sum_{n=0}^{L-1}q_{m}^{c,b}\left(n-\Delta^{\bar{c}}\right){\left(q_{m,ref}^{\bar{c},\bar{b}}\left(n-m\right)\right)}^{*}\\ =\frac{1}{L}\sum_{n=0}^{L-1}\left(\sum_{w_{0}=0}^{L-1}\sum_{\delta_{0}=1}^{L-1}s_{w_{0}}^{}s_{w_{0}+\delta_{0}}^{}e^{-j2\pi w_{0}\left(n+{d^{\prime }}_{c}\right)/L}e^{j2\pi \left(w_{0}+\delta_{0}\right)\left(n+{d^{\prime }}_{b}\right)/L}\right)\\ \;\;\;\;\;\times \left(\sum_{w_{2}=0}^{L-1}\sum_{\delta_{1}=1}^{L-1}s_{w_{2}}^{}s_{w_{2}+\delta_{1}}^{}e^{j2\pi w_{2}\left(n+{d^{\prime }}_{\bar{c}}\right)/L}e^{-j2\pi \left(w_{2}+\delta_{1}\right)\left(n+{d^{\prime }}_{\bar{b}}\right)/L}\right) \end{array} \end{array}$$

In addition, % denotes the modulo operation. The “shift and add property” of the m-sequence states that multiplication of an m-sequence with its own cyclic shift results in another m-sequence [37]. Here, $D_{\delta }$ denotes the amount of shift caused by the multiplication of an m-sequence by its shifted version with an offset δ ranging from 1 to L−1. Thus, (16) can be simplified as

(17) $$\begin{array}{c} \begin{array}{c} \sum_{n=0}^{L-1}q_{m}^{c,b}\left(n-\Delta^{\bar{c}}\right){\left(q_{m,ref}^{\bar{c},\bar{b}}\left(n-m\right)\right)}^{*}\;\;\;\;\;\;\;\;\;\;\;\;\;\\ =S_{\left({d^{\prime }}_{c}-{d^{\prime }}_{b}\right)}^{}\frac{1}{L}\sum_{n=0}^{L-1}\left(\sum_{\delta_{0}=1}^{L-1}e^{j2\pi \left(\delta_{0}\right)\left({d^{\prime }}_{b}\right)/L}e^{j2\pi D_{\delta_{0}}\left({d^{\prime }}_{c}-{d^{\prime }}_{b}\right)/L}e^{j2\pi \left(\delta_{0}\right)\left(n\right)/L}\right)\;\;\;\;\\ \;\times \left(S_{\left({{d^{\prime }}_{\bar{b}}}_{c}-{d^{\prime }}_{\bar{c}}\right)}^{}\right)\left(\sum_{\delta_{1}=1}^{L-1}e^{-j2\pi D_{\delta 1}\left({d^{\prime }}_{\bar{c}}-{d^{\prime }}_{\bar{b}}\right)/L}e^{-j2\pi \left(\delta_{1}\right)\left({d^{\prime }}_{\bar{b}}\right)/L}e^{-j2\pi \left(\delta_{1}\right)\left(n\right)/L}\right) \end{array} \end{array}$$

Equation (17) is simplified as

(18) $$\begin{array}{c} \sum_{n=0}^{L-1}q_{m}^{c,b}\left(n-\Delta^{\bar{c}}\right){\left(q_{m,ref}^{\bar{c},\bar{b}}\left(n-m\right)\right)}^{*}=S_{\left({d^{\prime }}_{c}-{d^{\prime }}_{b}\right)}^{}\left(S_{\left({{d^{\prime }}_{\bar{b}}}_{c}-{d^{\prime }}_{\bar{c}}\right)}^{}\right)\underset{\mu }{\underset{︸}{\sum_{\delta_{0}=1}^{L-1}e^{j2\pi D_{\delta_{0}}\left(\alpha \right)/L}e^{-j2\pi \left(\delta_{0}\right)\left(\beta \right)/L}}} \end{array}$$

where $\alpha ={d^{\prime }}_{c}-{d^{\prime }}_{b}+{d^{\prime }}_{\bar{b}}-{d^{\prime }}_{\bar{c}}$ and $\beta ={d^{\prime }}_{\bar{b}}-d_{b}^{\prime }$. When no STO exists, the cyclic shifts, $d_{\bar{c}}$ and $d_{\bar{b}}$ are equal to $d_{c}$ and $d_{b}$, respectively, because $c=\bar{c}$ and $b=\bar{b}$. Thus, α=0 and β=0. Hence, $\left|\mu \right|$ is given by L−1. Therefore, when there is no STO $\left(\Delta^{\prime }=0,d_{c}^{\prime }=d_{c}\right)$, the autocorrelation value is given by

(19) $$\begin{array}{c} \left|\sum_{n=0}^{L-1}q_{m}^{\bar{c},\bar{b}}\left(n-\Delta^{\bar{c}}\right){\left(q_{m,ref}^{\bar{c},\bar{b}}\left(n-m\right)\right)}^{*}\right|=\left(L^{2}-1\right)/L^{2} \end{array}$$

Moreover, in the presence of an STO, the received m-BTS is changed to another sequence with different $d_{c}^{\prime }$. In this case, its correlation value should be found through cross-correlation operation, which is derived as follows. In (14), $Y_{m-BTS}$ represents the interference term when the received signal has the same CID but a different BID $\left(c=\bar{c},b\neq \bar{b}\right)$. Using the “shift and add property” [37] of the m-sequence, the inter-beam interference can be expressed as

(20) $$\begin{array}{c} \left|\sum_{n=0}^{L-1}q_{m}^{\bar{c},b}\left(n-\Delta^{\bar{c}}\right){\left(q_{m,ref}^{\bar{c},\bar{b}}\left(n-m\right)\right)}^{*}\right|=\frac{1}{L}s_{\left(d_{c}-d_{b}\right)}^{\Delta^{\bar{c}}}\left(s_{\left(d_{\bar{b}}-d_{\bar{c}}\right)}^{m}\right)\mu . \end{array}$$

Because α≠β, ${\left|\mu_{\alpha =0,\alpha \neq \beta }^{}\right|}^{2}$ is given by

(21) $$\begin{array}{c} \begin{array}{c} {\left|\mu_{\alpha =0,\alpha \neq \beta }^{}\right|}^{2}=\sum_{\delta_{0}=1}^{L-1}\sum_{{\bar{\delta }}_{0}=1}^{L-1}e^{j2\pi (D_{\delta_{0}}-D_{{\bar{\delta }}_{0}})\alpha /L}e^{-j2\pi (\delta_{0}-{\bar{\delta }}_{0})\beta /L}\\ \;\;\;\;=\sum_{\delta_{0}=1}^{L-1}\left\{1+\sum_{{\bar{\delta }}_{0}=1,{\bar{\delta }}_{0}\neq \delta_{0}}^{L-1}e^{j2\pi (D_{\delta_{0}}-D_{{\bar{\delta }}_{0}})\alpha /L}e^{-j2\pi (\delta_{0}-{\bar{\delta }}_{0})\beta /L}\right\}. \end{array} \end{array}$$

As ${\left({\bar{\delta }}_{0}-\delta_{0}\right)}_{\%L}$ can be represented by τ ranging from 1 to L−1, ${\left|\mu_{\alpha =0,\alpha \neq \beta }^{}\right|}^{2}$ can be expressed as

(22) $$\begin{array}{c} {\left|\mu_{\alpha =0,\alpha \neq \beta }^{}\right|}^{2}=L-1+\left(L-2\right)\sum_{\tau =1}^{L-1}e^{j2\pi \tau \beta /L}=1 \end{array}$$

Using (22), (18) can be simplified as

(23) $$\begin{array}{c} \left|\sum_{n=0}^{L-1}q_{m}^{\bar{c},b}\left(n-\Delta^{\bar{c}}\right){\left(q_{m}^{\bar{c},\bar{b}}\left(n-m\right)\right)}^{*}\right|=\left(L+1\right)/L^{2} \end{array}$$

In (14), $\Lambda_{m-BTS}$ represents the interference term caused by cross-correlation when the received signal has a different CID $\left(c\neq \bar{c}\right)$. Using the “shift and add property” [37] of the m-sequence, the inter-cell interference term can be expressed as

(24) $$\begin{array}{c} \begin{array}{c} \left|\sum_{n=0}^{L-1}q_{m}^{c,b}\left(n-\Delta^{c}\right){\left(q_{m,ref}^{\bar{c},\bar{b}}\left(n-m\right)\right)}^{*}\right|=\frac{1}{L}s_{\left(d_{c}-d_{b}\right)}^{\Delta^{c}}\left(s_{\left(d_{\bar{b}}-d_{c}\right)}^{m}\right)\mu \end{array} \end{array}$$

As α≠0 and α≠β, ${\left|\mu_{\alpha \neq 0,\alpha \neq \beta }^{}\right|}^{2}$ is given by

(25) $$\begin{array}{c} \begin{array}{c} {\left|\mu_{\alpha \neq 0,\alpha \neq \beta }\right|}^{2}=L-1+\sum_{\tau =1}^{L-1}e^{j2\pi \tau \beta /L}\left\{\underset{\upsilon }{\underset{︸}{\sum_{\delta_{0}=1,\delta_{0}\neq L-\tau }^{L-1}e^{j2\pi (D_{\delta_{0}}-D_{{\left(\delta_{0}+\tau \right)}_{\%N}})\alpha /L}}}\right\} \end{array} \end{array}$$

Here, τ is a constant integer ranging from 1 to L−1. The values of variables $\delta_{0}$ and $D_{\delta_{0}}$ also range from 1 to L−1. Furthermore, L−τ is not considered for $\delta_{0}$ in (25) because it generates an out of range value. Replacing ${\left(D_{\delta }-D_{\delta +\tau }\right)}_{\%L}$ with $\tilde{D}$, *v* can be expressed as

(26) $$\begin{array}{c} \begin{array}{c} \upsilon =\sum_{\tilde{D}=1,\tilde{D}\neq L-\tau }^{L-1}e^{j2\pi \tilde{D}\alpha /L}\\ \;\;=\sum_{\tilde{D}=0}^{L-1}e^{j2\pi \tilde{D}\alpha /L}-e^{j2\pi 0\alpha /L}-e^{j2\pi (L-\tau )\alpha /L} \end{array} \end{array}$$

Because α is an integer, the first and second terms in (26) become zero and one, respectively. The third term can be rewritten as $e^{j2\pi (L)\alpha /L}e^{j2\pi (-\tau )\alpha /L}$. Because α is an integer, the first term becomes one. Thus, *v* can be simplified as

(27) $$\begin{array}{c} \upsilon =-1-e^{-j2\pi \tau \alpha /L} \end{array}$$

Using (23), (25), and (27), the inter-cell interference term in (24) is given by

(28) $$\begin{array}{c} \left|\sum_{n=0}^{L-1}q_{m}^{c,b}\left(n-\Delta^{c}\right){\left(q_{m,ref}^{\bar{c},\bar{b}}\left(n-m\right)\right)}^{*}\right|=\left\{\begin{array}{c} \frac{{\left(L+1\right)}^{3/2}}{L^{2}},\left(\alpha \neq 0,\beta \neq 0\right)\&\left(\alpha \neq \beta \right)\\ \frac{\left(L+1\right)}{L^{2}},\;\;\left(\beta =0\right)\&\left(\alpha \neq \beta \right) \end{array}\right. \end{array}$$

### 3.3. Examples

**Figure 3:**
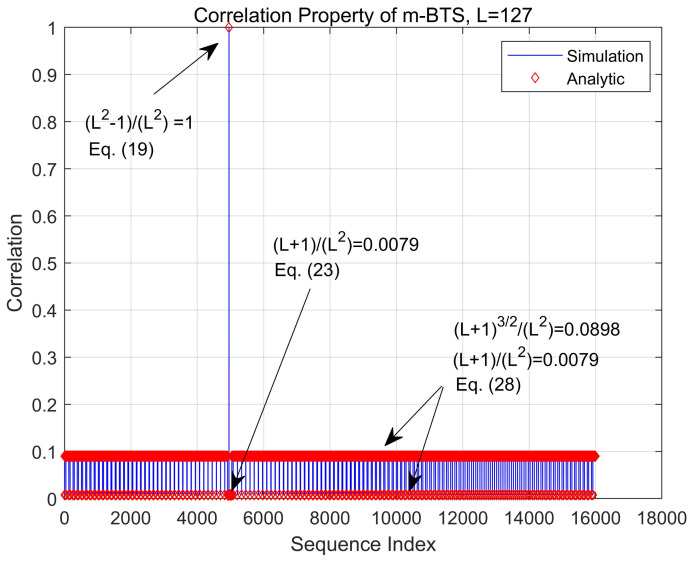
Correlation property of the m-BTS (STO = 0).

Figure 3 presents an example of the correlation function of the m-BTS when L=127. The analytic results derived in (19), (23), and (28) are compared with the simulation results when the STO is zero. Here, the CID and BID are set to 39 and 3 $\left(d_{\bar{c}}=39,d_{\bar{b}}=3\right)$, respectively. According to Figure 3, the maximum correlation peak occurs at the correct position, i.e., 4956 $\left(\left(d_{\bar{c}}\times N_{c}\right)+d_{\bar{b}}\right)$. As given in (19), autocorrelation value $\left(L^{2}-1\right)/L^{2}$ is close to one. The term $\left(L+1\right)/L^{2}$, corresponding to the inter-beam interference in (23), is 0.0079. Moreover, ${\left(L+1\right)}^{3/2}/L^{2}$ and $\left(L+1\right)/L^{2}$, corresponding to the inter-cell interference in (28), are 0.0898 and 0.0079, respectively. The results are indistinguishable in Figure 3 because the analytic and simulation results are approximately identical.

The maximum cross-correlation values of the ZC-BTS and m-BTS are compared for three different values of the sequence length (L) in Table 1. Here, the GS is included for comparison purposes. Table 1 shows that the maximum cross-correlation values of the ZC-BTS and m-BTS are significantly smaller than those of the GS for the three cases. The maximum cross-correlation value of the ZC-BTS is slightly smaller than that of m-BTS. As discussed in Section 3.1, the number of available sequences in the ZC-BTS decreases as the maximum STO value increases. In the ZC-BTS, the value of cyclic shift spacing must be increased to avoid the ambiguity condition in (9) as the maximum STO value increases. When the sequence length is 127 and the maximum STO is 30 samples, the number of available IDs in the ZC-BTS is 533 $\left(\left\lfloor \left(L-1\right)\times \frac{L}{z_{\max}}\right\rfloor \right)$. In addition, the m-BTS experiences an ambiguity problem because the received m-BTS is changed to another sequence with different $d_{c}$ in the presence of the STO $\left(\Delta^{\bar{c}}-m\neq 0\right)$. The peak is shifted in proportion to the product of the sequence length and STO value. To avoid the ambiguity problem, the sequences that can be obtained by the peak shift phenomenon in the presence of the STO should not be selected. Therefore, the number of available IDs in the m-BTS is reduced by the factor of the maximum STO value $\Delta_{\max}^{\prime }$. Under the same conditions $\left(\Delta_{\max}^{\prime }=30\right)$, the number of available IDs in the m-BTS is 533 $\left(\left\lfloor \frac{N^{d_{c}}}{{\Delta^{\prime }}_{\max}}\times N^{d_{b}}\right\rfloor \right)$. Here, $N^{d_{c}}\left(127\right)$ and $N^{d_{b}}\left(126\right)$ denote the maximum number of shifts for the CID and BID generations, respectively. However, the number of available IDs in the ZC-BTS and m-BTS can be increased by assigning IDs in the ambiguity region to spatially separated cells (not adjacent cells).

## 4. Simulations

In this section, performance of the proposed beam training technique for IRS-assisted cellular systems is evaluated via numerical simulations. The simple IRS-assisted cellular system model, shown in Figure 1, was used for simulation. It was assumed that the link between the BS and MS was blocked because of some obstacle, and the MS received a signal through the BS-IRS-MS link. The channel between BS/IRS and IRS/MS was assumed to experience Rician fading, which consisted of an LOS path and a non-LOS (NLOS) path. The k-factor was set to 15 dB. The NLOS path was generated by the spatial channel model (SCM) and composed of 20 rays with an azimuth spread of 2° [38,39]. The BS, IRS, and MS were assumed to have URAs with 16, 32, and four antenna elements, respectively. The continuously variable phase shifters are used for beamforming. The simulation parameters are listed in Table 2.

**Figure 4:**
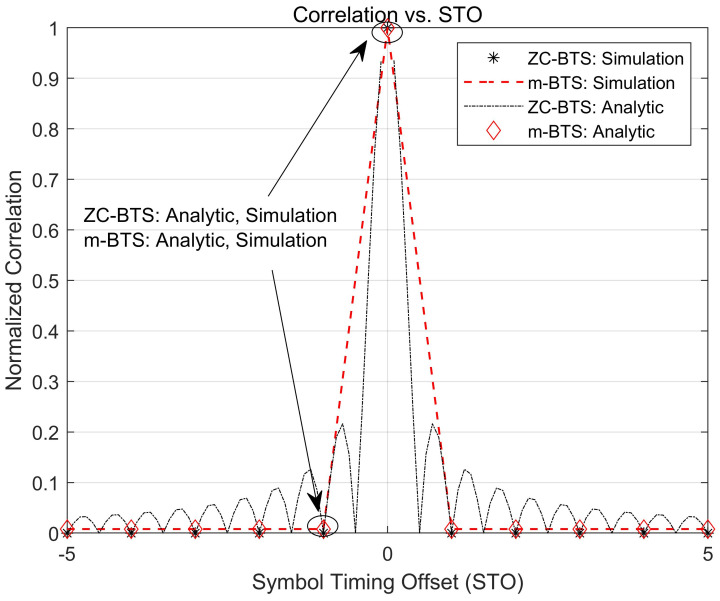
Correlation property of the ZC-BTS and m-BTS in the presence of the STO.

Figure 4 presents the correlation values of the ZC-BTS and m-BTS when the STO exists. According to (5), the autocorrelation of the ZC-BTS is maximum when the STO is zero. The correlation decreases in the sinc-like pattern. The autocorrelation becomes zero when the STO value is an integer greater than zero. According to (19), the autocorrelation of the m-BTS is maximum $\left(\frac{L^{2}-1}{L^{2}}\right)$ when the STO is zero. In the presence of the STO, the m-BTS is changed into another sequence $\left(\Delta^{\prime }\neq 0,{d^{\prime }}_{c}=d_{c}+\Delta^{\prime }L\right)$. In this case, the correlation value is given by $0.0079\left(\frac{L+1}{L^{2}}\right)$ according to (28). Moreover, the simulation results for the ZC-BTS and m-BTS overlap with the analytic results at the integer value of STO.

**Figure 5:**
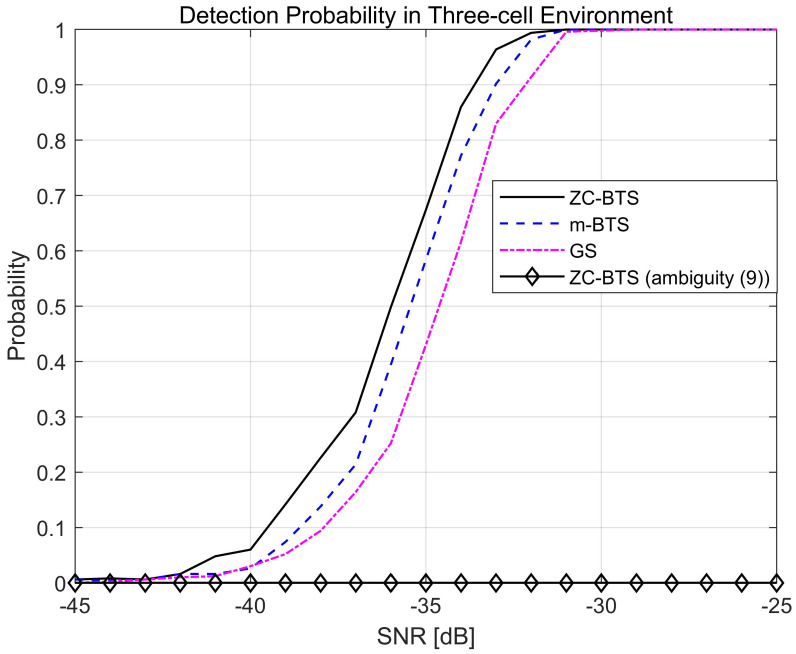
Detection probability for three BTSs.

Figure 5 compares the detection probabilities of the ZC-BTS and m-BTS when the system is synchronized. The detection probability is obtained by correlating the received signal with the reference BTS and finding the index with the maximum correlation value. If the detected CID and BID are correct, then the detection is declared “successful”. The simulation is performed in a three-cell environment. The MS is assumed to receive reflected signals from the IRS when the serving BS transmits the training signals. The training signals transmitted from two adjacent BSs are considered as interference. The lengths of the sequences (ZC-BTS, m-BTS, and GS) are set to 127. The other parameters used in the simulation are listed in Table 2.

Figure 5 shows that the ZC-BTS performs approximately 1 dB better than m-BTS. The m-BTS performs approximately 1 dB better than the GS. This is because the maximum cross-correlation values are on the order of the ZC-BTS (lowest), m-BTS, and GS (highest). A high detection probability is obtained in the low SNR region because of the beamforming gains of the BS (12 dB), IRS (15 dB), and MS (6 dB). Figure 5 also shows the detection probability of the ZC-BTS when the STO exists and the ambiguity condition in (9) is met. For the simulation, the root index, cyclic shift spacing, reference BID, and STO are set to $r^{c}$ = 54, z = 6, $\bar{b}$ = 5, and ($\Delta^{\bar{c}}-$ m) = 30, respectively. In this case, BID 5 is transmitted; however, the maximum correlation occurs at BID 10, resulting in a detection error.

**Figure 6:**
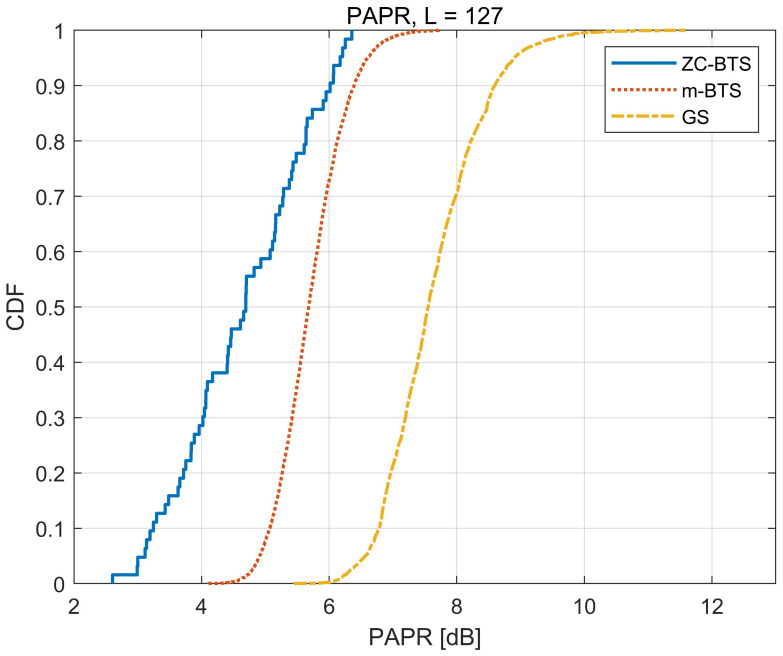
PAPR for three BTSs.

Figure 6 compares the PAPRs of the ZC-BTS, m-BTS, and GS. The ZC-BTS has the lowest PAPR because the ZC-BTS is generated using the ZC sequence with a constant envelope. The PAPR of m-BTS is higher than that of ZC-BTS; however, it is significantly lower than that of GS.

**Figure 7:**
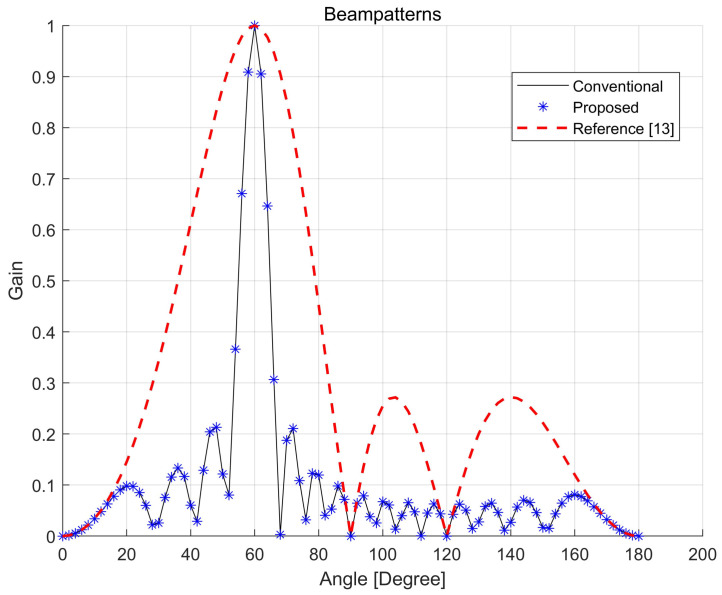
Comparison of beam patterns.

Figure 7 compares the beam patterns in the azimuth angle for three different techniques (conventional, proposed, and reference [30]) when the same number of antenna elements is used in the horizontal domain. Figure 7 shows that the proposed technique produces a beam pattern similar to conventional beamforming. The beam pattern in the proposed technique is sharper than that in the reference [30] because the URA in the proposed technique is divided into ULAs consisting of all antenna elements in the azimuth domain, as shown in Figure 2. However, the IRS elements in [30] are divided into multiple subarrays, resulting in wider beams.

**Figure 8:**
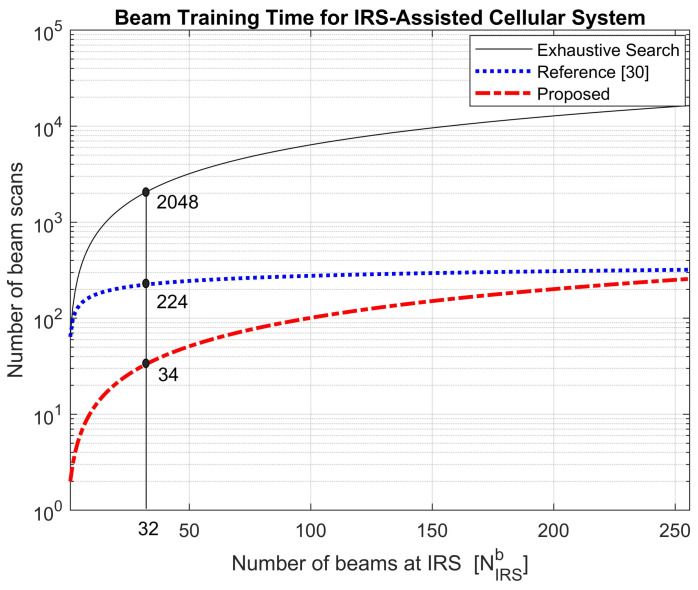
Number of beam scans required for IRS-assisted cellular systems.

Figure 8 compares the beam training time required for the BS-IRS-MS link when three different techniques (exhaustive search, proposed, and reference [30]) are used. The beam training time is compared in terms of the number of beam scans required for the entire BS-IRS-MS link. The number of beam scans required for an exhaustive search is given by $N_{BS}^{b}\times N_{IRS}^{b}\times N_{IRS}^{b}$. In [30], beam training is proposed for the IRS-MS link, assuming that the best beam pair of the BS-IRS link has already been found. For comparison with the proposed technique, an exhaustive search is assumed for the BS-IRS link. The number of beam scans required for the proposed technique is given by $u\times \left(v+N_{IRS}^{b}\right)\times w$, as discussed in Section 2. Figure 8 is plotted on a logarithmic scale with the parameters listed in Table 2. Figure 8 shows that the proposed technique with the ZC-BTS or m-BTS can significantly reduce the number of beam scans. For example, when the number of beams at the IRS is 32, the exhaustive search, reference [30], and proposed technique require 2048 $\left(16\times 32\times 4\right)$, 224 $\left(16\times 3.5\times 4\right)$, and 34 $\left(1\times \left(2+32\right)\times 1\right)$ beam scans, respectively.

## 5. Conclusions

In this paper, a fast beam training technique for IRS-assisted mmWave cellular systems with URAs is proposed to detect the best beam pairs of the BS-IRS-MS link simultaneously. Two different types of BTSs (ZC-BTS and m-BTS) were proposed to distinguish simultaneously transmitted beams from the BSs in a multi-cell multi-beam environment. The correlation properties of the ZC-BTS and m-BTS with STOs were analyzed in multi-cell multi-beam environments. It was revealed that the maximum cross-correlation value of the ZC-BTS is slightly smaller than that of the m-BTS. However, the maximum cross-correlation values of the ZC-BTS and m-BTS are significantly smaller than those of the GS. Both ZC-BTS and m-BTS experience an ambiguity problem when the STO exists. Consequently, in the proposed technique, the CID and BID should be selected to avoid ambiguity in the adjacent cells, considering the maximum STO value. In addition, it was also demonstrated that the ZC-BTS has a lower PAPR than the m-BTS. However, the m-BTS has a significantly lower PAPR than the GS. Finally, it was demonstrated that the proposed technique with the ZC-BTS and m-BTS can significantly reduce the number of beam scans without affecting the beam resolution in IRS-assisted mmWave cellular systems.

## Figures and Tables

**Table 1:** Maximum correlation values of the ZC-BTS, m-BTS, and GS.

	Maximum Correlation	*L* = 31	*L* = 63	*L* = 127
ZC-BTS	$1/\sqrt{L}$	0.1796	0.1260	0.0887
m-BTS	${\left(L+1\right)}^{3/2}/L^{2}$	0.1886	0.1289	0.0897
GS	$\frac{1+2^{\left\lfloor \frac{l+2}{2}\right\rfloor }}{L}$	0.2903	0.2698	0.1339

**Table 2:** Simulation parameters.

Simulation Parameter	Value
Channel model	Spatial channel model (SCM)
Antenna elements at BS $\left(N_{BS}^{A}\right)$	16
Beams at BS $\left(N_{BS}^{b}\right)$	16
Subarrays at BS $\left(N_{BS}^{s}\right)$	16
Reflect elements at IRS $\left(N_{IRS}^{A}\right)$	32
Beams at IRS $\left(N_{IRS}^{b}\right)$	32
Subarrays at IRS $\left(N_{IRS}^{s}\right)$	32
Antenna elements at MS $\left(N_{MS}^{A}\right)$	4
Beams at MS $\left(N_{MS}^{b}\right)$	4
Subarrays at MS $\left(N_{MS}^{s}\right)$	4
Antenna spacing at BS, IRS, and MS	λ/2
Sequence length (L)	127

## Data Availability

Not applicable.
